# Serum Cotinine versus Parent Reported Measures of Secondhand Smoke Exposure in Rural Appalachian Children

**DOI:** 10.13023/jah.0101.03

**Published:** 2019-04-30

**Authors:** Samrat Yeramaneni, Kimberly Yolton, Kurunthachalam Kannan, Kim N. Dietrich, Erin N. Haynes

**Affiliations:** HCA Research Institute, Sarah Cannon, samrat.yeramaneni@gmail.com; Cincinnati Children's Hospital Medical Center, Cincinnati, Ohio, kimberly.yolton@cchmc.org; University of Albany, Albany, New York, kurunthachalam.kannan@health.ny.gov; University of Cincinnati, Cincinnati, Ohio, kim.dietrich@uc.edu; University of Kentucky, Lexington, Kentucky, erin.haynes@uky.edu

**Keywords:** rural, cigarette, survey, community health, passive smoking, biomarkers

## Abstract

**Background:**

Secondhand smoke (SHS) exposure in Appalachian children and associated adverse effects is understudied and not well documented. This study assessed the prevalence of SHS exposure in Appalachian children by parental self-report and internal biological measure.

**Methods:**

SHS exposure was determined in children residing in rural Appalachian communities during their participation in the Communities Actively Researching Exposure Study between 2009 and 2013. Parents reported the number of smokers in the household and number of cigarettes smoked/day. Children ages 7–9 provided a serum sample for cotinine analysis. Parent reported measures and child serum cotinine measures of SHS exposure were compared with national and Appalachian-state estimates. Data analysis for the study was done in 2013.

**Results:**

Approximately 37% parents reported at least one smoker in the home, yet 50% of children had a detectible level of cotinine in serum. The mean serum cotinine level in children was 0.7 + 1.6 ng/mL. In homes of at least one reported smoker, an average of 20 cigarettes were smoked//day. Compared to 7.6% children, aged 3–19 years, exposed to SHS nationally, 36.6% children in our study were exposed to SHS living in Appalachian counties.

**Implications:**

Children living in rural Appalachian counties are significantly exposed to SHS exposure. Parental self-reports of smoking underestimates child exposure to SHS as measured by serum cotinine levels. Developing risk communication messages and implementing culturally appropriate interventions aimed at reducing tobacco dependence in rural Appalachian regions should be explored.

## INTRODUCTION

Secondhand smoke (SHS) is an important public health hazard, especially in children and adolescents.^1^ Assessment of SHS exposure by self-report versus measures of serum cotinine, a metabolite of nicotine, often yields inconsistent prevalence estimates. Based on parent-report measures, approximately 4.8 million children (≤12 years) in the United States (U.S.) are exposed to SHS in their homes.^2^ While the Centers for Disease Control and Prevention (CDC) reported 53.6% (or ~36 million) children (3–11 years), are exposed to SHS based on serum cotinine measures.^1^ Biological markers of exposure, such as serum cotinine, are superior to self-reported measures, as they better quantify exposure and minimize misclassification bias.^3^–^6^

Prevalence of tobacco use in the Appalachian Region is reportedly higher compared to other regions in the U.S. For instance, 12% of adults living in states with Appalachian counties self-reported smoking indoors with children present, compared to 8% in states with no Appalachian counties.^7^ However, this study did not investigate the surveyed population’s actual place of residence as being an Appalachian versus non-Appalachian state.^7^ Thus, the purpose of this study was to fill these gaps in the literature by determining the prevalence of SHS exposure in 7–9 year old Appalachian children using parent reports and serum cotinine measures. In addition, the current study evaluated SHS exposure prevalence in our cohort by comparison with national and state estimates. This study is important because it provides SHS prevalence data using biomarker data rather than self-report for Appalachian children.

## METHODS

### Study Participants

This study used data from the Community Actively Researching Exposure Study (CARES), a community-based participatory research partnership to address community concerns regarding manganese exposure.^8^ Participants of the CARES cohort reside in Marietta and Cambridge, Ohio, and their surrounding areas. Eligibility for participation included children aged 7, 8, and 9 years that have resided in the catchment areas throughout their life with no plans to move for at least one year. In addition, biological mothers must have resided in the catchment area during pregnancy. All participants signed informed consent and assent forms approved by the Institutional Review Board of the University of Cincinnati. Study participants were enrolled between 2009 and 2013. SHS exposure was assessed by (1) parental self-reports of smoking and (2) child serum cotinine levels. Data for this study was analyzed in 2013.

### Parental Self-Report

A parent/legal guardian from each household was asked to report the number of smokers residing in the household, their relationship to the child, and the number of cigarettes smoked/day. Based on a reported response of smoking at least one cigarette/day by the parent/legal guardian,^9^ we classified parental self-reported smoking as *“yes/no”*, and number of smokers in the household as *“0”*, *“1”*, and *“≥2”*. Total number of cigarettes smoked/day/household was calculated by summing the number of cigarettes smoked by each member in the household. A child was considered exposed to SHS if a household member reported smoking ≥1 cigarette/day.^10^

### Child Serum Cotinine Analysis

Whole blood was collected into 10-mL tubes and samples were allowed to clot at room temperature for 15–25 minutes before centrifugation to obtain serum. Approximately 2 mL of serum, clear and free of red cells, was transferred to cryovials labeled with a subject study code number before shipping to Wadsworth Center, New York State Department of Health for analysis. Serum cotinine levels were determined using techniques consistent with the standardized isotope dilution liquid chromatography/ tandem mass spectrometry (LC/MS/MS) method used in the National Health and Nutrition Examination Survey (NHANES).^1^ A child was considered exposed to SHS if their serum cotinine levels were ≥0.05 ng/mL.

### Statistical Analysis

Age of participants was presented as mean ± standard deviation (SD), all other continuous or discrete variables were presented as medians (interquartile range, IQR), frequencies, or percentages as appropriate. Serum cotinine levels were log-transformed due to skewed distribution. Approximately 51% (n=162) of the samples in our study were below the level of detection (0.05 ng/mL), however, reported actual values were used for statistical analyses. SHS exposure prevalence in our study cohort were compared to (1) national estimates using NHANES 2007–2008 data^1^ and (2) estimates from total states containing Appalachian counties (n=13), and Ohio, using the 2007 National Survey of Children’s Health (NSCH) data.^11^ A two-tailed *P*-value of <0.05 was used to judge statistical significance. All analyses were performed using SAS 9.3 version (SAS Institute Inc., Cary NC).

## RESULTS

A total of 404 children were recruited for the study. Table 1 summarizes demographic characteristics of the cohort and SHS exposure estimates. Approximately 37% of parents reported at least one smoker in the home. Twenty-five percent of homes had one smoker, whereas 12% had two or more smokers. Of the homes with reported smokers, 63% of children have a mother who reported smoking, 50% of the children have a father who reported smoking, and 28% of the children have other family members who reported smoking. These categories are not exclusive.

Based on serum cotinine levels (≥0.05 ng/mL), 49% of the children in the CARES cohort were exposed to SHS. Of the participants with available serum cotinine measures (n=320), the mean serum cotinine level was 0.7 ± 1.6 ng/mL and the range was 0.001–10.9 ng/mL. There was no statistically significant difference in the average serum cotinine level between boys and girls (boys; 0.79 ± 1.77 ng/mL and girls; 0.55 ± 1.38 ng/mL, *P*=0.17).

Table 2 compares the prevalence estimates of SHS exposure in the CARES cohort to national estimates and estimates from states containing Appalachian counties. Based on parent reported measures, 37% of children in the CARES cohort, 15% of children nationally, 9.7% of children in states containing Appalachian counties, and 13.2% of children in Ohio were exposed to SHS. Based on serum cotinine levels, prevalence of SHS exposure in the CARES cohort (49%) was similar to national estimates (50%).

## DISCUSSION

The prevalence of SHS exposure among children aged 7–9 years living in the CARES catchment area was found to be approximately 37% based on parental self-report and 50% based on child serum cotinine measures. Self-reported measures due to their inherent limitations of recall bias, over/under reporting, withholding information, and cultural appropriateness often result in data that may inaccurately represent exposure-outcome relationship leading to biased risk estimates.^3^ Internal dose markers of exposure are superior to self-reported measures as they better quantify exposure and thereby minimize misclassification bias.^12^ Cotinine, a metabolite of nicotine, measures body burden of the exposure and is considered a valid and reliable marker of exposure to tobacco smoke.^13^ The prevalence of SHS exposure, as measured by serum cotinine, in the nonsmoking population decreased significantly from 52.5% during 1999–2000 to 40.1% in 2007–08 as reported by the CDC from the 1999– 2008 National Health and Nutritional Examination Survey (NHANES).^1^ Yet, several reports indicate the decline in SHS exposure rates being lower for children (4–11 years) and adolescents (12–19 years) compared to adults.^14^,^15^ Our findings are consistent with the national estimate of 50% of children exposed to SHS based on serum cotinine levels of ≥0.05 ng/mL and support the literature that biological markers are superior to self-reported measures in truly estimating exposure levels.

Prevalence of SHS exposure, based on parent self-reported measures, in children ≤17 years was 12% in states containing Appalachian counties and 16% in Ohio. In contrast, children in the CARES cohort had a much higher prevalence of SHS exposure. This may be attributed to the higher prevalence of tobacco use in Appalachian counties compared to non-Appalachian counties.^16^ Researchers who have studied the role of cultural significance on tobacco use in Appalachian communities reported that Appalachians, compared to non-Appalachians, are not only attracted to the use of tobacco products socially, but also believe it is important for their economic survival.^16^ This finding reiterates the importance of the development of culturally-appropriate public health interventions targeted towards Appalachians. Given the significant health consequences of secondhand tobacco smoke exposure on children’s health, these interventions are sorely needed.

Socioeconomic status may not be the only determinant that is playing a role in the increased prevalence of smoking in Appalachian communities. Availability of tobacco cessation programs and tobacco control policies may also be crucial in decreasing adult smoking in the rural Appalachia. Studies have shown that increases in the price of cigarettes^17^–^19^ and stronger smoke-free laws^18^, ^20^, ^21^ are associated with lower levels of adult smoking. It is interesting to note that the Clean Indoor Air (CIA) ordinances, which ban smoking in workplaces, restaurants, and bars, is particularly strong in Ohio unlike other states containing Appalachian counties, such as Alabama, Georgia, Kentucky, Mississippi, South Carolina, and West Virginia, that have weak statewide CIA laws^22^ Yet, the high prevalence of SHS exposure in children from the CARES cohort compared to national estimates and other Appalachian states raises some concerns. First, it is important to measure the implementation and coverage of CIA laws in Appalachian regions where smoking prevalence among adults is high, education levels are low, and poverty is higher compared to non-Appalachian regions. Ferketich et al.^22^ observed that less than 20% of the 332 Appalachian communities surveyed in six Appalachian states had adopted a comprehensive workplace, restaurant, or bar ordinance. Furthermore, communities with higher unemployment and lower levels of education were less likely to have a strong ordinance. Although this study did not measure the strength and coverage of CIA ordinance in our catchment areas, it may be possible that children were exposed to SHS in public places where there was less restriction to smoking. In contrast, presence of stronger CIA ordinance laws in our study sites may exclude the possibility of children’s exposure to SHS in public places. It is highly likely that the primary source of SHS exposure in our study cohort was from homes and/or automobiles, as the percentage of children in our study population residing in a home with at least one smoker (25%) is much higher than the national estimate of 18.2% children, aged 3–11 years, living in households where someone smoked cigarettes inside their home.^1^In addition, the young age of our study population makes them more likely to spend time in their homes where smoking restrictions are voluntary and may be limited.^23^

Parents play an important role in children’s health and future smoking habits. Kabir et al., reported that ‘voluntary’ household smoking restrictions resulted in 20%–50% reduction in childhood SHS exposure. In this systematic review article Kabir et al., identified that both self-reported and biological measures were utilized to estimate SHS exposure in children aged 0–17 years.^24^ Longitudinal studies have shown that children of parents who smoke in households tend to initiate smoking early, continue smoking in later life, and be heavy consumers of tobacco products.^25^ Children who are exposed to smoking in cars are more likely to be open to smoking in the future and have a higher risk of being current smokers,^26^ reiterating the fact that children should be protected from SHS not only in their homes but also inside vehicles. Early and active involvement of parents in educational interventions for smoking-related behaviors is essential. Interventions should include intensive counseling to parents to reduce tobacco dependence initiated at pediatricians and family physicians’ clinics. A novel intervention consisting of providing measurements of indoor air quality and motivational interviewing to mothers who smoked indoors proved to be effective in changing their smoking behavior.^27^ Currently there is no evidence that at any one intervention is entirely effective in reducing parental smoking and children’s exposure to SHS. A comprehensive approach that includes counseling and education at home, offices, and primary care clinics and regulatory and economic policies should be adopted.^28^

This study has a few limitations. The questionnaire did not ascertain the location of smoking (i.e., inside the home, outside only, automobiles, or in the presence of their child) or the duration of exposure (i.e., number of hours spent in the same location where smoking was occurring). Due to lack of available data on serum cotinine levels in children from studies conducted in other Appalachian states, we are only able to compare the prevalence estimates using parent reported measures.

## IMPLICATIONS

Findings from this study support existing evidence that children are at increased risk of SHS exposure and that SHS exposure remains an important public health issue, especially in rural Appalachian communities. Future research on SHS exposure should include a more detailed characterization of tobacco exposure. There is also a need to explore risk communication messages and effective intervention strategies geared towards smoking cessation in rural Appalachian communities.

**SUMMARY BOX**

**What is already known about this topic?** Children exposed to secondhand smoke (SHS) are at increased risk for premature death and varied illnesses. Stricter smoke-free policies in workplaces and public places have reduced SHS exposure. However, smoking within households continues to place children and adolescents at high risk of SHS exposure. There is insufficient information on the range of SHS exposure in children, especially living in rural Appalachian regions.

**What is added by this report?** This study quantified the levels of SHS exposure in children aged 7–9 years living within households with smokers in rural Appalachian regions. Based on self-reported parent measures 37% of children were exposed to SHS. However, using serum cotinine measures approximately 50% of children were found to be exposed to SHS. Measuring SHS exposure using internal dose biomarkers, such as serum cotinine, provides an unbiased estimation of exposure not subject to the reporting bias as observed in survey data. Children living in rural Appalachian regions are exposed to much higher levels of SHS exposure compared to children living in non-Appalachian regions.

**What are the implications for public health practice, policy, and research?** Culturally appropriate education interventions, counseling, and motivational interviewing to reduce tobacco dependence, initiated at their primary clinics, should be implemented. Families should be encouraged to adopt smoke-free policies inside their households and inside vehicles.

## Figures and Tables

**Table 1:** Demographic characteristics of the study cohort and SHS exposure measures

Demographic Characteristics (n=404)	
Age in years [Mean ± SD]	8.4 ± 0.9
Gender [n (%)]	
Boys	217 (53.7)
Girls	187 (46.3)
Race/Ethnicity [n (%)]	
Caucasian^a^	379 (93.8)
Hispanic^b^	6 (1.5)
Other	19 (4.7)

**Parental Smoking Measures (n=404)**	

Parental self-reported smoking [n (%)]	
Yes	148 (36.6)
No	256 (63.4)
Smokers in the household [n (%)]	
0	256 (63.4)
1	101 (25.0)
>2	47 (11.6)
Median (IQR^c^) cigarettes smoked/day/household	*20.0 (10.0, 30.0)*

**Child Serum Cotinine (n=320)**	

Median (IQR)	0.05 (0.02, 0.42)
≥0.05 ng/mL	158 (49.4)
<0.05 ng/mL	162 (50.6)

a Non-Hispanic white.

b Hispanic ethnicity regardless of race.

c IQR: interquartile range

**Table 2:** Prevalence of SHS exposure in CARES cohort, national estimates, and estimates from states containing Appalachian counties

National Estimates (2007–2008)^1^		Estimates from States containing Appalachian Counties (2007–2008)^6^			CARES Cohort (2009–2013) (7–9 years)

			Total States	Ohio	

Age group (years)	Parent self-reported measures (%)	Age group (years)	Parent self-reported measures (%)	Parent self-reported measures (%)	Parent self-reported measures (%)
3–11	14.6	0–11	9.7	13.2	
12–19	15.2	12–17	16.3	22.2	36.6
3–19	7.6	0–17	11.9	16.2	
